# Developing New Cyclodextrin-Based Nanosponges Complexes to Improve Vitamin D Absorption in an In Vitro Study

**DOI:** 10.3390/ijms24065322

**Published:** 2023-03-10

**Authors:** Francesca Uberti, Francesco Trotta, Pasquale Pagliaro, Daniel Mihai Bisericaru, Roberta Cavalli, Sara Ferrari, Claudia Penna, Adrián Matencio

**Affiliations:** 1 Laboratory of Physiology, Department of Translational Medicine, University of Piemonte Orientale, Via Solaroli 17, 28100 Novara, Italy; francesca.uberti@med.uniupo.it (F.U.); sara.ferrari@uniupo.it (S.F.); 2 Dipartimento di Chimica and NIS, Università di Torino, Via P. Giuria 7, 10125 Torino, Italy; daniel.bisericaru@edu.unito.it (D.M.B.); adrian.matencioduran@unito.it (A.M.); 3 Laboratory of Cardiovascular Physiology, Dipartimento di Scienze Cliniche e Biologiche, Università Degli Studi di Torino, Regione Gonzole 10, 10043 Orbassano, Italy; pasquale.pagliaro@unito.it (P.P.); claudia.penna@unito.it (C.P.); 4 Dipartimento di Scienza e Tecnologia del Farmaco, Università di Torino, Via P. Giuria 9, 10125 Torino, Italy; roberta.cavalli@unito.it

**Keywords:** Vitamin D, nanosponge, intestinal cells, absorption mechanism

## Abstract

Vitamin D plays an important role in numerous cellular functions due to the ability to bind the Vitamin D receptor (VDR), which is present in different tissues. Several human diseases depend on low vitamin D3 (human isoform) serum level, and supplementation is necessary. However, vitamin D3 has poor bioavailability, and several strategies are tested to increase its absorption. In this work, the complexation of vitamin D3 in Cyclodextrin-based nanosponge (CD-NS, in particular, βNS-CDI 1:4) was carried out to study the possible enhancement of bioactivity. The βNS-CDI 1:4 was synthesized by mechanochemistry, and the complex was confirmed using FTIR-ATR and TGA. TGA demonstrated higher thermostability of the complexed form. Subsequently, in vitro experiments were performed to evaluate the biological activity of Vitamin D3 complexed in the nanosponges on intestinal cells and assess its bioavailability without cytotoxic effect. The Vitamin D3 complexes enhance cellular activity at the intestinal level and improve its bioavailability. In conclusion, this study demonstrates for the first time the ability of CD-NS complexes to improve the chemical and biological function of Vitamin D3.

## 1. Introduction

Vitamin D comprises the isoforms D3 (found in humans) and D2 (found in plants). Vitamin D3 (VitD3) plays an important role in numerous cellular functions not only at the bone level, but also at other levels such as the cardiovascular one. VitD3 is able to induce its effects through binding to the vitamin D receptor (VDR), which is present in different tissues, including endothelium, vascular smooth muscle, and cardiomyocytes. The importance of VitD3 in the cardiovascular system is proven by the observation that the deletion of VDR from the heart results in hypertrophy and contributes to the acceleration of atherosclerosis [1]. In recent years, it has been shown that a correct intake of VitD3 can avoid deficiencies in various pathological and non-pathological situations. Recent studies have also shown that VitD3 deficiency appears to be associated with more compromised and severe clinical stages of COVID infection. In this context, the supplementation of VitD3 could be a good strategy. However, VitD3 presents several problems of solubility and stability that prevent its final administration. To solve this problem, one possibility is its complexation with Cyclodextrins (CDs). CDs are truncated cone-shaped oligosaccharides made up of α-(1,4) linked glucose units, obtained from the degradation of starch by the enzyme Cyclodextrin Glucosyltransferase (CGTAse) [2]. Although both inorganic and organic salts and neutral molecules can form complexes with CDs, they are more generally used to complex poorly soluble drugs or bioactive compounds, creating so-called “inclusion complexes” [3]. In general, the inclusion complex formation improves the apparent solubility of the drug, which increases its final concentration, even affecting the structure and possibly its bioactivities. However, other effects such as increasing stability, controlled release or chiral separation occur when the inclusion complex is formed [4,5].

The CDs have demonstrated their ability to form an inclusion complex with VitD3 [6]. However, for different applications, some CD-based materials present better characteristics, e.g., slower release and higher stability of the drug. Among them, the so-called CD-based Nanosponge (CD-NS) is an innovative cross-linked polymer possessing good swelling properties and with a three-dimensional network and modulable tunable structure such as crystalline, amorphous or spherical structure [7]. In recent years, it has been proposed as a possible drug carrier to be considered in pharmaceutical applications [8], as it is stable, insoluble, biocompatible and capable of encapsulating drugs through the formation of inclusion and non-inclusion complexes. In addition, nanosponges have been reported to be able to increase the stability of drugs and reduce their degradation [9]. Recent reviews [7,10] point to their wide potential and minimal negligible toxicity [11,12], increasing their bio-capacities in several applications including the ability to (i) increase or improve the apparent solubility of poorly soluble drugs, (ii) modulate the drug release and activity, (iii) protect drugs against several agents, (iv) enhance bioactivities, (v) absorb contaminants or deliver the drug, etc. 

In the last few years, several drugs and macromolecules, such as nutraceuticals or proteins, have improved their stability and effectiveness when complexed with CD-NS [7]. So, considering these evidence, it is reasonable to increase VitD3 bioactivities in complex form.

Bearing the above in mind, the aim of this work was to analyze the improvement of bioavailability of VitD3/CD-NS complexes compared to other VitD3 forms. Specifically, it was complexed with bNS-CDI 1:4, one of the most used for biological applications. After the chemical analysis, several biological effects were investigated to exclude cytotoxicity or intestinal irritability maintaining the correct intestinal barrier functions necessary to improve the vitD3 absorption.

## 2. Results

### 2.1. Complexation of Vitamin D on βNS-CDI (1:4)

**Figure 1:**
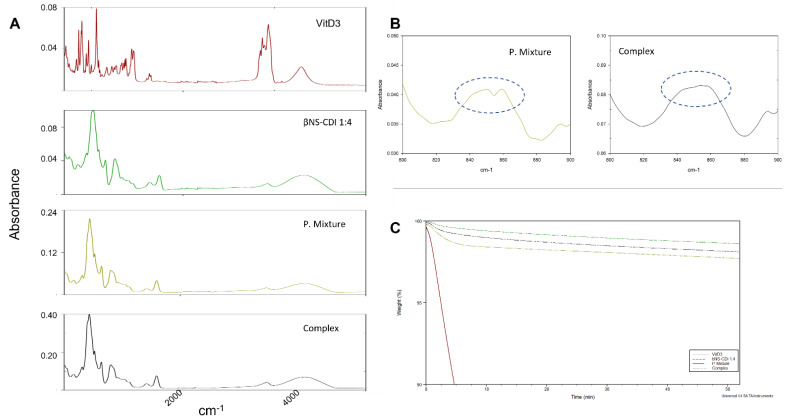
(**A**) FTIR–ATR spectra of VitD3, βNS–CDI (1:4), physical mixture and complex. (**B**) detail of 800–900 cm^−1^ spectra, indicated with dotted circle, peaks at 859 and 843 cm^−1^. (**C**) Isothermal TGA of VitD3, βNS–CDI (1:4) (called bNS-CDI 1:4), physical mixture (P.Mixture) and complex at 200 °C.

VitD3 presents an unstable nature that prevents the use of some complexation techniques (e.g., mixing in water for 24 h). The kneading process presents some advantages because it is scalable, easy and efficient [13]. The 5% of loading (1:0.05 *w/w* ratio) is considerably lower than several complexes prepared [7,12] suggesting (i) that all the VitD3 will enter completely in the cavities and (ii) the high quantity of βNS-CDI (1:4) will control the release equilibrium. Figure 1A shows the FTIR-ATR spectra of the VitD3, βNS-CDI (1:4), physical mixture and inclusion complex. VitD3 showed the typical peaks around 2850–3000 cm^−1^ (free CH3 stretching); 1750 cm^−1^ (ester stretching); or 1160 cm^−1^ (carbonyl stretching) [14]. The occurrence of the carbonate bond peak at 1739 cm^−1^ in the FTIR spectrum is the characteristic feature of NS [15] An interesting change in the peak appearance was found around 850 cm^−1^ (Figure 1B, -C-H vibration). To better understand the complex formation and the stability of the complex, an isothermal TGA was performed at 200 °C (temperature where VitD3 started the degradation in TGA, data not showed). The results are in Figure 1C, showing that VitD3 is degraded quickly while βNS-CDI (1:4) is kept practically intact. A higher decrease in the mass of the physical mixture was shown in comparison with the complex (1.3 times in 60 min).

### 2.2. Analysis of Different Forms of Vitamin D3 in a Time-Course Study

**Figure 2:**
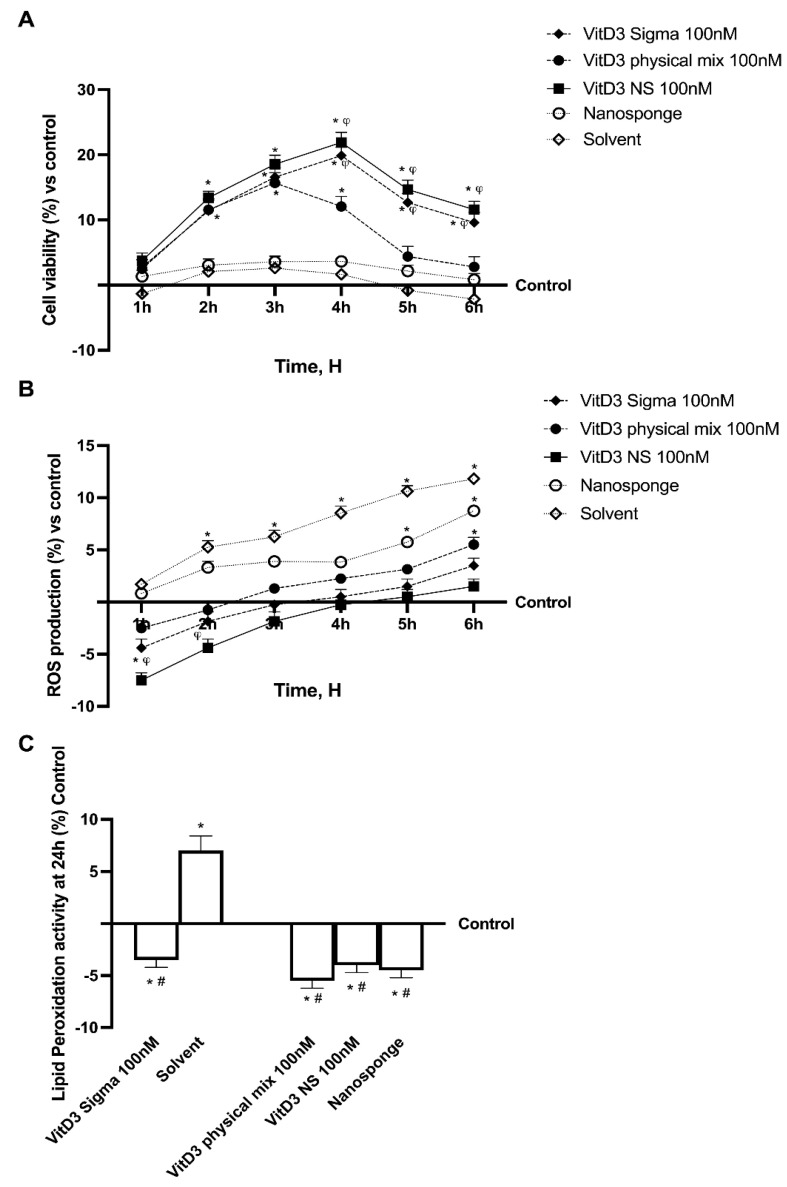
Analysis of different forms of VitD3 in time-dependent study. (**A**) Mitochondrial Metabolism, (**B**) ROS production and (**C**) Lipid Peroxidation analysis. Data are expressed as mean ± SD (%) of 5 independent experiments normalized to control. (**A**,**B**) * *p* < 0.05 vs. control; ^φ^ *p* < 0.05 vs. VitD3 physical mix 100 nM (**C**) * *p* < 0.05 vs. control (untreated cells); ^#^ *p* < 0.05 vs. Solvent (ethanol).

Before analyzing the permeability and transport mechanism of Vitamin D3-BCDI 1:4 loaded nanosponge (called VitD3 NS), a CaCo-2 cell line was used to perform a time-dependent study to rule out any cytotoxic effects. The analysis was performed by comparing the effects of VitD3 NS with Physical Mix VitD3-BCDI 1:4 (called VitD3 Physical Mix), BCDI (1:4 nanosponge, called nanosponge) and reference VitD3 (called VitD3 Sigma) by testing them at the same concentration on cell viability and radical oxygen species (ROS) production in a time-course study (from 1 to 6 h). Cell viability results, measured by the 3-(4,5-Dimethylthiazol-2-yl)-2,5-diphenyltetrazolium bromide (MTT) assay, showed a time-dependent trend of all substances (Figure 2A), demonstrating that the beneficial effects were maintained during all periods of treatment excluding any cytotoxic effect (*p* < 0.05 vs. control). In particular, the cells treated with VitD3 NS 100 nM showed high viability compared to the control (*p* < 0.05) and compared to other forms of VitD3 tested (*p* < 0.05), suggesting that VitD3 NS 100 nM is not toxic to intestinal epithelial cells. Moreover, VitD3 NS 100 nM was shown to be well tolerated by intestinal cells, with a peak of cell viability at 4 h of treatment, compared to the VitD3 Physical Mix, and VitD3 Sigma at the same time point (about 45% and 10%, respectively, *p* < 0.05). Notably, the data obtained on the nanosponges alone and the solvent showed that they did not influence mitochondrial metabolism, maintaining values at the level of untreated cells with a slight, but not significant, reduction in cell viability at the end of the treatment. All these findings suggest that the molecules tested are not toxic to intestinal epithelial cells. However, additional experiments were carried out to confirm the well-being of the intestinal epithelium after treatment with VitD3 NS, by analyzing whether the tested substances could induce oxidative stress. For this reason, ROS production was evaluated on CaCo-2 cells from 1 to 6 h of treatment with VitD3 NS 100 nM, VitD3 Physical Mix 100 nM and VitD3 Sigma 100 nM. As shown in Figure 2B, none of the forms of VitD3 were able to increase ROS production, maintaining them at normal physiological conditions. Even if all the forms of VitD3 tested were able to maintain low ROS levels, the better effect was induced by VitD3 NS 100 nM (about 80% vs. VitD3 Physical Mix and 10% VitD3 Sigma, *p* < 0.05). Concurrently, a small amount of ROS production from nanosponges and VitD3 Sigma solvent (Figure 2B), although within physiological values, thus suggesting the absence of oxidative stress. Finally, to confirm the correct cellular function and avoid mitochondrial dysfunction at the end of the treatment, further experiments were carried out by analyzing the reduction in malonaldehyde (MDA) levels following VitD3 NS 100 nM, VitD3 Physical Mix 100 nM and VitD3 Sigma 100 nM treatment for 24 h. Therefore, as reported in Figure 2C, the data obtained demonstrated the same trend previously observed, confirming the security of VitD3 NS 100 nM use on intestinal epithelial cells (about 27% vs. VitD3 Physical Mix and 15% vs. VitD3 Sigma, *p* < 0.05).

### 2.3. Permeability Analysis of Different Forms of VitD3 Using an In Vitro Model of Intestinal Barrier

**Figure 3:**
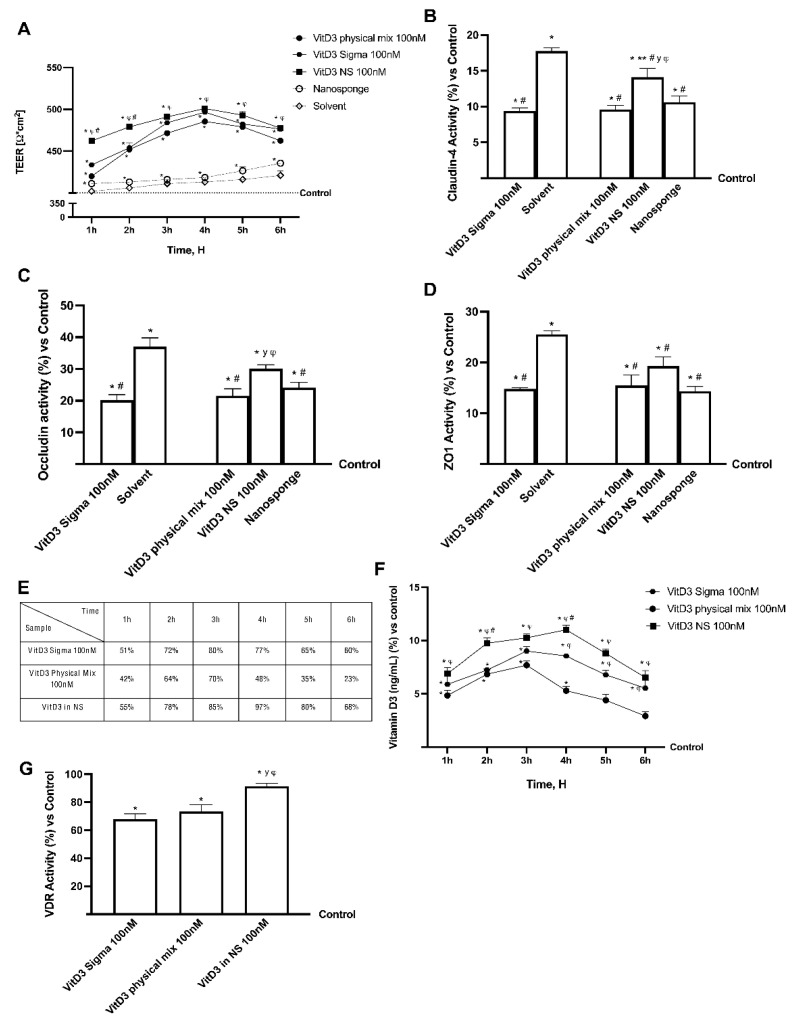
Permeability study on CaCo-2 cells. In (**A**) TEER (transepithelial electrical resistance) Value using EVOM3; from (**B**–**D**) the analysis of TJ measured by Enzyme-Linked Immunosorbent Assay (ELISA) test (Occludin, Claudin1, and ZO-1 (Zona occludens 1), respectively); in (**E**) the Papp (Apparent Permeability Coefficient) values in which data < 0.2 × 10^−6^ cm/s mean very poor absorption with a bioavailability < 1%, data between 0.2 × 10^−6^ and 2 × 10^−6^ cm/s with bioavailability between 1 and 90%, and data > 2 × 10^−6^ cm/s mean very good absorption with a bioavailability over 90%. In (**F**) VitD3 quantification measured by ELISA kit. In (**G**) Vitamin D3 Receptor activity measured by ELISA kit. Data are mean ± SD of five independent experiments performed in triplicates. From (**B**–**D**) means ± SD are expressed comparing data to control value (0% line) and all molecules result * *p* < 0.05 vs. control; ** *p* < 0.05 vs. nanosponge; ^#^ vs. solvent; ^y^ vs. VitD3 Physical Mix 100 nM; ^φ^ vs. VitD3 Sigma 100 nM. On the contrary, in (**A**,**E**,**F**), the control samples are specifically reported and VitD3 NS 100 nM, VitD3 Physical Mix 100 nM and VitD3 Sigma 100 nM are *p* < 0.05 vs. control. In conclusion, in (**G**) means ± SD are expressed comparing data to control value (0% line) and all molecules result * *p* < 0.05 vs. control; ^φ^ *p* < 0.05 vs. VitD3 physical mix 100 nM; ^#^ *p* < 0.05 vs. VitD3 sigma.

Further experiments were performed using a 3D in vitro model to mimic the in vivo intestinal barrier complexity to assess permeability and obtain additional information about the VitD3 NS intestinal absorption. In this context, the VitD3 NS, VitD3 Physical Mix and VitD3 Sigma at the same concentration were tested from 1 to 6 h in order to evaluate transepithelial electrical resistance (TEER) values, the apparent permeability coefficient (Papp) values, and the VitD3 concentration in order to analyze the bioavailability. The data reported in Figure 3 show that intestinal adsorption has a physiological trend; in particular, the passage through the intestinal epithelium demonstrates that all VitD3 forms were able to maintain the epithelial integrity increasing the ionic flux of the paracellular exchanges across the intestinal epithelial compared to the control (*p* < 0.05). Indeed, VitD3 NS 100 nM demonstrates a better effect than VitD3 Physical Mix 100 nM and VitD3 Sigma 100 nM during all treatment times (*p* < 0.05). Subsequently, TJ’s evaluation also confirmed these results; indeed, VitD3 NS 100 nM exerted the most significant effects compared to VitD3 Physical Mix 100 nM and VitD3 Sigma 100 nM regarding claudin-4 (32% and 34%, respectively, *p* < 0.05), occludin (28% and 33%, respectively, *p* < 0.05) and zonula occludens-1 (ZO-1, 20% and 23%, respectively, *p* < 0.05) and compared to control value (reported as 0 line, *p* < 0.05). From these promising results, which confirmed the correct functionality of the intestinal epithelium, further experiments were carried out by measuring the permeability rate, analyzing the flux of nonelectrolyte tracers (expressed as the permeability coefficient as reported) and the amount of VitD3 that crosses the intestinal barrier in order to reach the target site. 

The data obtained from the analysis of the basolateral environment (Figure 3E) confirmed our previous findings, as the amount of VitD3 NS 100 nM was able to release a huge amount of VitD3 with more efficiency over time than VitD3 Physical Mix 100 nM and VitD3 Sigma 100 nM (*p* < 0.05); in particular, VitD3 NS exerted a maximum effect at 4 h compared to the other forms of VitD3 (about 50.5% VitD3 Physical Mix and about 21% VitD3 Sigma, *p* < 0.05). In addition, data obtained from basolateral level quantification (Figure 3F) showed that VitD3 NS was able to cross the barrier and reach the plasma level in greater amounts than the control (*p* < 0.05) and the other forms of VitD3 (about 52% vs. VitD3 Physical Mix and about 22% vs. VitD3 Sigma, *p* < 0.05) with the greatest effects at 4 h. Finally, in order to confirm the biological activity of VitD3 NS, the modulation of VDR was investigated; as reported in Figure 3G, the VDR activity was increased after the treatment with three different forms of Vit3D compared to control (*p* < 0.05); in particular, the major activity was shown in the cells treated with VitD3 NS with an increase of 35% and 25% compared to VitD3 Sigma and VitD3 physical, respectively (*p* < 0.05). 

## 3. Discussion

Vitamin D deficiency is a global health issue affecting more than 1 billion children and adults worldwide [16]. This condition has been associated with many acute and chronic illnesses, including preeclampsia, childhood dental caries, periodontitis, autoimmune disorders, infectious diseases, cardiovascular diseases, various types of cancer, type 2 diabetes, and neurological disorders [17]. Since VitD3 is highly sensitive to light and oxygen, it is easily degraded. Consequently, deviations of label claims from actual amounts of Vitamin D in foods are common [18]. For this reason, especially in the elderly, exogenous administration through food supplements is necessary [19]. Despite its beneficial effects, Vitamin D administrations are restricted due to its poor bioavailability and very low aqueous solubility. Indeed, VitD3 is fat-soluble, and it requires lipid foods as a base for fortification, which is not compatible with consumer demand for low-fat foods [20]. Several studies showed that its absorption efficiency varies between 55% to 99%. However, it does not depend upon the fat content consumed with food. Nonetheless, lipid composition impacts Vitamin D bioavailability [19]. Actually, the absorption efficiency of Vitamin D is also increased by supplements in which Vitamin D is encapsulated into micelles, microcapsules, or liposomes. Emulsification of drugs or nutrients and their insertion into micelles or microcapsules have many benefits: higher stability to aggregation and gravitational separation; higher optical clarity; protection from degradation, light, and oxidation; and improved bioavailability of water-insoluble and difficultly absorbed compounds [19,21,22]. The innovative formulations by nano-complexation may include CDs, which, in this study, were used to complex and form the innovative vehicle. CDs are natural oligosaccharides widely used in many fields, such as biomedicine, cosmetics, food industry, wastewater remediation and catalysis. CDs have inclusion the capacity and ability to improve desired physico-chemical properties of specific molecules, such as apparent solubility and stability [7]. CDs insoluble polymers are called NS, referring to their sponge-like structure, since they have high porosity and capacity to entrap different types of molecules into the matrix. This study proved the successful embedding of Vitamin D3 in CD-NS, when equilibrated in aqueous solution. Indeed, differences can be found in the spectra comparing the physical mixture and the complex form. The FTIR-ATR spectrum of the physical mixture showed the superposition of FTIR-ATR peaks of both VitD3 around 850 cm^−1^ (-C-H vibration) to βNS-CDI (1:4) peaks, while in the complex, these peaks disappeared, indicating the interaction of VitD3 with CD-NS because of the encapsulation of drug. The encapsulation of drugs can cause the loss or displacement of characteristic peaks of the drug, as it happened and reports in the bibliography [23]. On the other hand, the thermostability study demonstrated a greater loss of VitD3 in the physical mixture, due to the non-formation of the inclusion complex. It is widely known that the formation of the inclusion complex is capable of protecting and increasing the stability of the drug inside it [12]. In addition, all these vitamin D forms were also tested mimicking oral human administration in vitro to explore their biological effectiveness. Cell metabolism, oxidative stress, and lipid peroxidation results showed that VitD3 NS is well tolerated by intestinal cells. In addition, the absorption study by the human intestinal 3D model demonstrated that oral administration is possible; indeed, bioavailability experiments indicated that orally administered VitD3 is effectively absorbed and distributed, exerting its biological functions acting on VDR. In particular, VitD3 NS has a high amount of VitD3 reaching the plasma level compared to the control and to other two forms of VitD3 with maximum absorption at 4 h, confirming the hypothesis that nanosponge encapsulation improves VitD3 bioavailability during the physiological time of intestinal digestion. In other words, the desired concentration in blood could be obtained more efficiently with the administration of the inclusion complex than free vitD3, for example the same concentration of VitD3 was achieved in 1 h with the complex, than in 3 h with the free drug (Figure 3F). In addition, VitD3 treatment exerts a role in improving permeability by enhancing tight junction proteins; actually, in epithelial cells, tight junction formation plays a key role in the intestinal barrier, and this is mediated by proteins such as claudin, occludin, and ZO-1 that are necessary for epithelial barrier activity. As expected, VitD3 NS has been shown to maintain epithelial integrity and ion exchanges across the intestinal barrier, suggesting that this complex is able to cross the cell monolayer without negatively altering the epithelium. Summarizing, these results support the idea that nanosponges are the best choice to improve the bioavailability of molecules with poor bioavailability, including VitD3. Therefore, the application of this novel form of a vehicle could be an excellent strategy to ameliorate pharmaceutical applications for poorly soluble active ingredients.

## 4. Materials and Methods

### 4.1. Synthesis of βNS-CDI 1: 4 by Mechanochemistry

The following procedure is already reported in the literature [15], with slight modifications. The cross-linked βCD Nanosponges were prepared using the ball mill (Planetary Ball Mill: PM200 High Speed Planetary Ball Mill, Retsch; Pedrengo BG, Italy) in a one-step reaction without solvents, respecting the 1:4 molar ratio between anhydrous cyclodextrin and carbonyl diimidazole: 3.75 g of β-CD (gift of Roquete Freres, Lestrem-France) were placed inside a 50 mL jar containing 10 zirconia balls. The amount of CDI (purchased from Sigma-Aldrich, Milano-Italy) added in each batch to maintain the 1:4 molar ratio was 2.25 g.

After 3 h of sun wheel rotation at 600 rpm, changing the direction from clockwise to anti-clockwise every 15 min, the reaction was completed. The measured external temperature of the jars was around 60 °C. After the synthesis a finely ground white/yellowish powder is obtained; subsequently, the powder is dispersed in water and washed several times with deionized water and acetone. All syntheses were extracted with Pressurized Solvent Extraction (PSE, SpeedExtractor E-914 from Buchi; Cornaredo MI, Italy), to remove the residual imidazole in the NS structure and unreacted material.

### 4.2. Vitamin D3 Complexation on βNS-CDI 1: 4 and Stability

The complex was prepared using a kneading approach to prevent eventual degradation. Briefly, 15 mg of VitD3 (Tocris Bioscience, Bristol-UK) were added in a mortar with 300 mg of βNS-CDI (1:4) to obtain a 5% of loading. 100 µL of 50/50 deionized H_2_O/EtOH were added to the sample and mixed for 90 min manually. The physical mixture was prepared mixing for 10 min at the same proportion, but without solvent. The powder was collected and dried in the air. The complex was confirmed using FTIR-ATR (PerkinElmer Spectrum 100 FT-IR Spectrometer; Waltham, MA, USA) with 16 scans.

To study the thermostability, a TGA was performed: VitD3, βNS-CDI, physical mixture and complex were subjected to thermal gravimetric analysis using a Q500 thermogravimetric analyzer (TA instruments, New Castle, DE, USA) from 30 to 200 °C at 20 °C/min, and after 60 min at 200 °C.

### 4.3. Agents Preparation

The formulation previously prepared was diluted to obtain a final VitD3 concentration of 100 nM [24]. VitD3 Physical Mix VitD3-BCDI 1:4 (once complexed, the βNS-CDI (1:4) is called BCDI 1:4) was prepared weighting 1 mg and dissolving it in 2 mL of medium and diluting it to obtain the final concentration to be tested in the well (100 nM). This concentration is reported in the literature to be the best concentration in a range from 1 to 100 nm [25,26,27]. Cyclodextrin was chosen to form the complex with vitamin D3 due to its good availability, low cost and single cavity size [28]. Additionally, it has the ability to form complexes with large number of molecules, it is non-toxic and has an excellent ability to capture organic and inorganic molecules, making it most suitable for our purpose [29]. VitD3 was prepared by weighing 1 mg and dissolving it in 2 mL of medium to have an initial concentration of 1.3 mM; this was diluted to obtain the final concentration of 100 nM. Reference VitD3 (Merck Life Science, Rome, Italy) was prepared by weighing 1 mg and dissolving it in 2 mL of ethanol 100% (Merck Life Science, Italy) and was diluted to obtain in the well the final concentrations to be tested (100 nM). 0.007% ET-OH was also tested alone to rule out vehicle interference.

### 4.4. Cell Culture

The human colorectal adenocarcinoma cell line CaCo-2 purchased from the American Type Culture Collection (ATCC), was cultured in Dulbecco’s Modified Eagle’s Medium/Nutrient F-12 Ham medium (DMEM/F12, Merck Life Science, Rome, Italy) mixed with 10% fetal bovine serum (FBS, Merck Life Science, Italy), 2 mM L-glutamine (Merck Life Science, Italy) and 1% penicillin/streptomycin (Merck Life Science, Italy) and maintained at 37 °C in an incubator at 5% CO_2_ and 95% humidity. The cells were used at passage numbers between 26 to 32 to preserve the physiological balance between paracellular permeability and transport properties [30]. Specifically, once 80% confluence was reached, the cells were seeded 1 × 10^4^ cells in 96-well plates to study cell viability by MTT; 1 × 10^6^ cells in 6-well plates to study the intracellular mechanisms involved by kit ELISA analysis and 1.8 × 10^4^ cells in a 24-well plate on 6.5 mm Transwell^®^ with 0.4 μm pore polycarbonate membrane insert (Merck Life Science, Italy) in a 24-well plate to perform the absorption study.

The human intestinal mucus-secreting cell line HT29-MTX, kindly donated by Molecular Pathology and NanoBioImaging of Professor Isidoro (Italy), was cultured in DMEM containing 10% FBS, 2 mM L-glutamine and 1% penicillin-streptomycin and maintained at 37 °C in a 5% CO_2_ incubator. The cells used for the experiments were at passages between 10 to 20 when maintaining the correct mucus-secreting phenotypes [31]. For the experiments, cells were plated at 1 × 10^4^ cells in a 96-well plate to verify the cell viability by MTT test; 1 × 10^6^ cells in 6-well plates to study the intracellular mechanisms involved and 2 × 10^3^ cells on 6.5 mm Transwell^®^ with 0.4 μm pore polycarbonate membrane insert (Merck Life Science, Italy) in a 24-well plate to explore the intestinal permeability. Cells plated on Transwell^®^ insert were maintained in a complete medium changed every other day, first basolaterally and then apically for 28 days before the treatment [32].

### 4.5. Cell Viability

The analysis of cell viability was performed using a classical technique based on the MTT-based In Vitro Toxicology Assay Kit (Merck Life Science, Rome, Italy) [33], following the manufacturer’s instructions. Indeed, at the end of treatment, the cells were incubated with 1% MTT dye for 2 h in an incubator at 37 °C, 5% CO_2_, and 95% humidity, and then the purple formazan crystals were dissolved in an equal volume of MTT Solubilization Solution. The absorbance was analyzed by spectrophotometer (Infinite 200 Pro MPlex, Tecan, Männedorf, Switzerland) at 570 nm with correction at 690 nm, and results were expressed compared to the control (0% line), which represented untreated cells. The results will represent the percentage of viable cells compared to the control, which will allow understanding whether the stimulation might be safe or not.

### 4.6. In Vitro Intestinal Barrier Model

**Figure 4:**
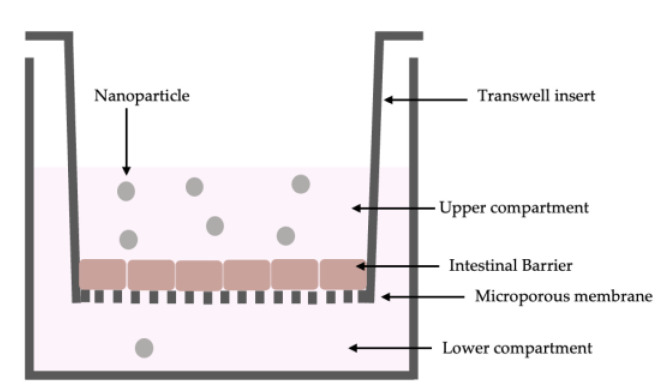
Schematic representation of in vitro Intestinal Barrier Model.

The CaCo-2 cells (enterocytes) were placed in co-culture with HT29-MTX cells (goblet cells) in a 9:1 ratio (Caco-2:HT29-MTX) [34]. This co-culture formed a functional epithelial barrier characterized by high electrical resistance and the presence of physiological intestinal properties such as transport mechanisms. This model (Figure 4) was made to analyze the passage through the intestinal barrier of the three different VitD3 formulations. For this reason, the transepithelial electrical resistance (TEER) values were determined with EVOM3, coupled with STX2 chopstick electrodes (World Precision Instruments, Sarasota, FL, USA) measuring every 2 days for 28 days until reaching a TEER value ≥ 400 Ωcm^2^ before the treatment [35,36]. The time required for the cell monolayer formation, cell differentiation, and intestinal villi maturation. Before treatment, the medium at the apical and basolateral environments was changed to create different pH conditions: pH around 6.5 at the apical level (acidic pH mimicking lumen of small intestine) and pH around 7.4 at the basolateral level (neutral pH mimicking human blood) [37]. The cells were kept for 15 min at 37 °C and 5% CO_2_, after that, the TEER values were measured again before the start of the experiment to verify the stabilization of the values. The cells were treated with three different VitD3 formulations for 2 h to 6 h before the successive analysis, including the permeability assay measured by Papp analysis [30]. Briefly, the Papp (cm/s) was calculated with the following formula of the cited paper:

- Papp = dQ/dt ⇥ 1/m0 ⇥ 1/A ⇥ V Donor
- dQ: amount of substance transported (nmol or μg);
- dt: incubation time (sec);
- m0: amount of substrate applied to donor compartment (nmol or μg);
- A: surface area of Transwell membrane (cm^2^);
- VDonor: volume of the donor compartment (cm^3^).
- Negative controls without cells were tested to exclude Transwell membrane influence.

### 4.7. ROS Production

The quantification of superoxide anion release was obtained following a standard protocol based on the reduction in cytochrome C [38], and the absorbance in culture supernatants was measured at 550 nm using the spectrophotometer (Infinite 200 Pro MPlex, Tecan, Männedorf, Switzerland). Specifically, 100 μL of cytochrome C (Merck, Milan, Italy) was added to all the wells, while 100 μL of superoxide dismutase (Merck, Milan, Italy) and 100 μL of cytochrome C were added to empty wells and the plate was then incubated for 30 min. After that, 100 μL was taken from each well and the absorbance was measured with a spectrophotometer (Infinite 200 Pro MPlex, Tecan, Männedorf, Switzerland) at 550 nm. The O_2_ rate was expressed as the mean ± SD (%) of nanomoles per reduced cytochrome C per microgram of protein compared to the control (0 line). 

### 4.8. Vitamin D Quantification

The competitive ELISA assay kit (FineTest) was performed to detect the metabolically active form of vitD, as reported in the literature [39]. In detail, at the end of each treatment, 50 μL of each sample was collected and immediately used. At each sample, 50 μL of biotin-detection and 100 μL of SABC working solution were added and incubated at 37 °C for 30 min. At the end, the supernatants were discarded, and then 90 μL TMB substrate plus and 50 μL of stop solution were added. Finally, the 96-well plate was analyzed by a spectrometer at 450 nm (Infinite 200 Pro MPlex, Tecan, Männedorf, Switzerland). In addition, it was necessary to plot a standard curve (range 1.563–100 ng/mL) including the background (zero well) to perform a quantification.

### 4.9. SOD Activity

The level of SOD was measured following the manufacturer’s instructions (Cayman’s Superoxide Dismutase Assay Kit; Tallinn, Estonia) which reveals all three types of SOD (Cu/Zn, Mn, and FeSOD). Briefly, in the standard well were added 200 µL of the Radical Detector and 10 µL of the Standard properly diluted; in the sample wells were added 200 µL of Radical Detector and 10 µL of the tested sample. After the addition of 20 µL of Xanthine Oxidase to all the wells, the plate was gently shaken and incubated for 30 min. Finally, the level of SOD present on 3D cell lysates was measured by comparing data to a standard curve (0.05–0.005 U/mL). The absorbance of all samples was measured through a spectrometer (Infinite 200 Pro MPlex, Tecan, Männedorf, Switzerland) at 440–460 nm and the results were expressed as means (%) compared to the control [38].

### 4.10. Lipid Peroxidation Activity

Lipid peroxidation in cells was estimated using the TBARS Assay Kit (Cayman Chemical, Tallinn, Estonia) [40]. Briefly, 100 μL of SDS solution was added to 100 μL of sample or standard. Then, 4 mL of Dye Reagent was added to each vial before boiling them for 1 h. After cooling down the samples for 10 min, the vials were centrifuged for 10 min at 1600× *g* at 4 °C and then 150 μL of samples or standard were added to the wells of a 96 multi well; the absorbance of all samples was measured through a spectrometer (Infinite 200 Pro MPlex, Tecan, Männedorf, Switzerland) at 530–540 nm. The concentration was compared to a standard curve (range 0–50 µM) and the results were then compared to the control (0% line), which represented untreated cells.

### 4.11. Occludin Quantification Assay

The Human Occludin (OCLN) ELISA kit (MyBiosource, San Diego, CA, USA) was used to measure the presence of occludin in the coculture lysates following the manufacturer’s instructions [30]. Briefly, the cells were lysed with cold phosphate-buffered saline (PBS) 1× and centrifuged at 1500× *g* for 10 min at 4 °C. A quantity of 100 μL of each sample was added to a strip well and incubated at 37 °C for 90 min; then, the supernatants were removed and 100 μL of Detection Solution A was added to each of them and incubated for 45 min at 37 °C. After this time, the wells were washed with Wash Solution and incubated with 100 μL of Detection Solution B for 45 min, and then 90 μL of Substrate Solution was added, incubating for 20 min at 37 °C in the dark. Finally, after adding 50 μL of Stop Solution, the plate was analyzed by a spectrometer (Infinite 200 Pro MPlex, Tecan, Männedorf, Switzerland) at 450 nm. The concentration was compared to a standard curve (range from 0 to 1500 pg/mL) and the results were then compared to the control (0% line), which represented untreated cells.

### 4.12. Claudin 1 Detection

The Human Claudin1 ELISA kit (MyBiosource, San Diego, CA, USA) was measured in the coculture lysates, following the manufacturer’s instructions [30]. Briefly, the cells were lysed with cold PBS 1× and centrifuged at 1500× *g* for 10 min at 4 °C. A quantity of 100 μL of each sample was added to a well and incubated at 37 °C for 90 min; then, the materials were removed and, to each well was added 100 μL of Detection Solution A, and incubated for 45 min at 37 °C. After this time the wells were washed and 100 μL of Detection Solution B in each well was added and then incubated for 45 min. Then, 90 μL of Substrate Solution in each well was also added and the plate was incubated for 20 min at 37 °C in the dark. A quantity of 50 μL of Stop Solution was used to stop the reaction and the plate was analyzed by a spectrometer (Infinite 200 Pro MPlex, Tecan, Männedorf, Switzerland) at 450 nm. The concentration was compared to the standard curve (range from 0 to 1000 pg/mL) and the results were then compared to the control (0% line), which represented untreated cells.

### 4.13. ZO-1 Detection

The Human Tight Junction Protein 1 (TJP1) ELISA kit (MyBiosource, San Diego, CA, USA) was used to measure the presence of tight junction protein 1 or Zona occludens 1 (ZO1) in cell lysates in the coculture, following the manufacturer’s instructions [41]. Briefly, the cells were lysed using trypsin and then collected by centrifugation. Then, cells were washed three times with cold PBS 1× and then resuspended in PBS 1×; subsequently, the cells were subjected to ultrasonication four times and then they were centrifuged at 1500× *g* for 10 min at 4 °C in order to remove cellular debris. A quantity of 100 μL of each sample was added to a well and incubated at 37 °C for 90 min; then, the materials were removed, and to each well was added 100 μL of Detection Solution A and incubated for 45 min at 37 °C. Wells were washed with Wash Solution and after 100 μL Detection Solution B was added to each well. After incubation of 45 min, the wells were washed again and 90 μL of Substrate Solution was added in each well and then incubated for 20 min at 37 °C in the dark. Finally, 50 μL of Stop Solution was added and then the plates were read by a spectrometer (Infinite 200 Pro MPlex, Tecan, Männedorf, Switzerland) at 450 nm. The concentration was compared to the standard curve (range from 0 to 1000 pg/mL) and the results were then compared to the control (0% line), which represented untreated cells.

### 4.14. VDR Activity

The Human Vitamin D Receptor (VDR) ELISA kit (MyBiosource, San Diego, CA, USA) was used to measure the presence of VDR in cell lysates of the coculture, following the manufacturer’s instructions. Briefly, the cells were lysed using trypsin and then collected by centrifugation. Then, cells were washed three times with cold PBS 1× and then resuspended in PBS 1×; subsequently, the cells were subjected to ultrasonication four times and then they were centrifuged at 1500× *g* for 10 min at 4 °C to remove cellular debris. A quantity of 100 μL of each sample was added to a well and incubated at 37 °C for 90 min; then, the materials were removed, and to each well was added 100μL of Detection Solution A and incubated for 45 min at 37 °C. Wells were washed with Wash Solution and after 100 μL Detection Solution B was added to each well. After incubation of 45 min, the wells were washed again and 90 μL of Substrate Solution was added in each well and then incubated for 20 min at 37 °C in the dark. Finally, 50 μL of Stop Solution was added and then the plates were read by a spectrometer (Infinite 200 Pro MPlex, Tecan, Männedorf, Switzerland) at 450 nm. The concentration was expressed as ng/mL and the data were compared with the standard curve (range 0.625 ng/mL–40 ng/mL).

### 4.15. Statistical Analysis

For each experimental protocol, at least four independent experiments have been carried out; the results are expressed as means ± SD of independent experiments performed on four technical replicates. One-way ANOVA followed by the Bonferroni post hoc test was used for statistical analysis, and pairwise differences were compared by Mann–Whitney U tests followed by Welch’s test. *p* values < 0.05 were considered statistically significant.

## 5. Conclusions

In conclusion, this study demonstrates for the first time the ability of CD-NS complexes to improve the thermostability, chemical and biological function of VitD3. In particular, the chemical stability confirms the security of in vitro study in intestinal cells. This supports the potential of the VitD3 complex to be a new dietary supplementation. Although the in vitro data are very clear and promising, in vivo or even human studies would be needed to confirm these observations before assuming an absolute efficacy of this Vitamin D CD-NS. Thus, the results of the present study about the effectiveness in improving VitD3 absorption may support the hypothesis that oral administration in humans can be considered a valid therapeutic strategy to obtain beneficial therapeutic effects under low VitD3 conditions.

## Data Availability

Raw data are preferably deposited at the Laboratory of Physiology UPO and at the Dept. Di Chimica IFM University of Turin, ensuring appropriate measures so that raw data are retained in full forever under a secure system. The data presented in this study are available on reasonable request from the corresponding author.

## Abbreviations

ANOVA	Analysis of Variance
ATCC	American Type Culture Collection
CD-NS	Cyclodextrin-based nanosponge
CDs	Cyclodextrins
CGTAse	Cyclodextrin Glucosyltransferase
DMEM	Dulbecco’s Modified Eagle’s Medium
DMEM/F12	Dulbecco’s Modified Eagle’s Medium/Nutrient F-12 Ham medium
FBS	fetal bovine serum
MTT	3-(4,5-Dimethylthiazol-2-yl)-2,5-diphenyltetrazolium bromide
NS	Nanosponge
OCLN	Occludin
Papp	Apparent permeability coefficient
PBS	Phosphate Buffered Saline
PSE	Pressurized Solvent Extraction
ROS	Radical oxygen species
SDS	Sodium dodecyl sulfate
SOD	Superoxide Dismutase
TBARS	Thiobarbituric acid reactive substances
TEER	Transepithelial electrical resistance values
TGA	Thermal gravimetric analysis
TJ	Tight Junction
TJP1	Human Tight Junction Protein 1
VDR	Vitamin D Receptor
VitD3	Vitamin D3
VitD3 NS	Vitamin D3-BCDI 1:4 nanosponge
ZO-1	Zona occludens 1
